# Iatrogenic aortic dissection after minimally invasive aortic valve replacement: a case report

**DOI:** 10.1186/s13019-016-0531-y

**Published:** 2016-08-24

**Authors:** Mohamed Ehab Ramadan, Lamia Buohliqah, Juan Crestanello, James Ralston, David Igoe, Hamdy Awad

**Affiliations:** 1 Department of Anesthesiology, The Ohio State University, Wexner Medical Center, N411 Doan Hall, 410 West 10th Avenue, Columbus, OH 43210 USA; 2 Department of Otolaryngology - Head & Neck Surgery, The Ohio State University, Wexner Medical Center, Columbus, OH USA; 3 Department of Surgery, Division of Cardiac Surgery, The Ohio State University, Wexner Medical Center, Columbus, OH USA; 4 Perfusion Services, The Ohio State University, Wexner Medical Center, Columbus, OH USA

**Keywords:** Case Report, Iatrogenic aortic dissection, AVR, PCI, TEVAR, TAVR

## Abstract

**Background:**

As minimally invasive cardiac and vascular procedures are on the rise, the incidence of iatrogenic acute aortic dissection (IAAD) will increase. Cardiovascular professionals should be aware about the risk factors, means of prevention and best management options for IAAD in the perioperative setting.

**Case presentation:**

We present the successful clinical management of a complicated case of IAAD after minimally invasive aortic valve replacement.

**Conclusion:**

High index of suspicion is required for prompt diagnosis of IAAD; collaboration of the whole perioperative team is imperative for management of this catastrophe.

## Background

Iatrogenic acute aortic dissection (IAAD) is a fatal complication that can occur after open cardiac surgery, complex percutaneous coronary intervention (PCI), thoracic endovascular aortic aneurysm repair (TEVAR) and transaortic valve replacement (TAVR). As minimally invasive procedures are on the rise, the incidence of IAAD will increase. IAAD carries high mortality and represents a huge challenge to all cardiovascular professionals including cardiac surgeons who encounter this clinical scenario [1]. Here we present the successful clinical management of a case of IAAD after minimally invasive aortic valve replacement (AVR). We will review the literature for incidence, risk factors, diagnosis and management of IAAD in open cardiac surgery, PCI, TEVAR and TAVR.

## Case presentation

**Fig. 1:**
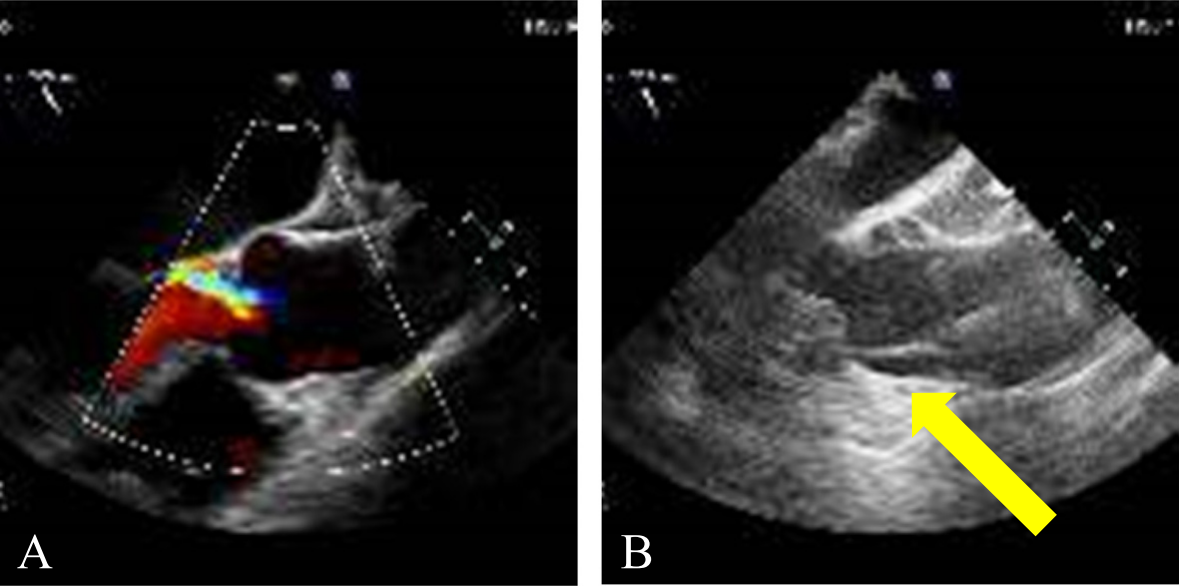
Diagnosis of IAAD by TEE. TEE Mid Esophageal Ascending Aorta Long Axis view. **a** Before aortic cannulation; **b** After aortic cannulation showing intimal flap on the anterior aortic wall at the sinotubular junction with the development of true and false aortic lumens and extending antegrade and retrograde: Type A acute aortic dissection (Yellow Arrow)

Sixty-seven years old female patient with history of atypical chest pain, exertional dyspnea, asthma and hypothyroidism was diagnosed with aortic insufficiency. Her preoperative echocardiography findings were dilated aortic root (3.7 cm), mild left ventricular hypertrophy and ejection fraction (70 %). Evaluation by cardiac catheterization revealed severe aortic regurge and normal coronary arteries. An 8 cm median sternotomy was performed. The distal ascending aorta was cannulated at blood pressure of 100 mmHg systolic and cardiopulmonary bypass (CPB) was initiated with good flow and pressures in the cannula. The surgeon noticed a hematoma around the cannulation site. With the aid of Transesophageal echocardiography (TEE) done by the anesthesiologist, diagnosis of a type A acute aortic dissection was made, that was extending to the aortic root and the descending aorta (Fig. 1). CPB was terminated without any significant event, and right common femoral artery was cannulated for alternate route of CPB. The sternotomy incision was extended to a full sternotomy. Cooling for 48 min to 18 degrees Centigrade nasopharyngeal temperature was achieved for deep hypothermic circulatory arrest. Bentall procedure with a composite Carpentier-Edwards Perimount 23 mm tissue valve and a 28 mm Hemashield graft was performed. Circulation was resumed and patient was rewarmed. Both coronary arteries buttons were attempted but failed due to frail intima at the coronary ostia. Left main coronary artery was reimplanted via a Cabrol procedure with an 8 mm Hemashield graft. Bypass of the right coronary artery with reverse saphenous vein graft was also done. We weaned the patient from CPB but there was progressive dilatation of the right ventricle and left ventricular dysfunction, so we were concerned with the graft for the left main coronary artery. We went back on CPB to perform bypasses to the left anterior descending artery and the obtuse marginal artery using reverse saphenous vein grafts. Then, we weaned the patient from CPB again with difficulty on high doses of inotropic support. Due to long CPB time (336 minutes), the team decided to support the patient with venoarterial extracorporeal membrane oxygenation (VA ECMO) by re-cannulating the right common femoral artery and vein. The chest was left open at the end of the procedure due to cardiac edema and distension. The patient was transferred to the ICU in extremely critical condition. The cross-clamp time was 70 min and circulatory arrest time was 17 min. The patient required massive blood transfusion (140 units of blood products) in the perioperative period. The chest wall was closed 3 days postoperative and was kept on ECMO support for 17 days. The patient had a vigorous postoperative course complicated by cardiac tamponade that required multiple bedside washouts. She also suffered from subacute right middle cerebral artery territory infarct that developed intraoperative with residual left sided hemiparesis. She also had prolonged intubation due to failure of multiple spontaneous breathing trials and she required tracheostomy on the twentieth postoperative day. Her last echocardiography findings before discharge were normal right and left ventricular sizes with mild to moderate systolic dysfunction (Ejection fraction: 30–35 %). The patient was discharged on the 35th postoperative day and she is continuing her rehabilitation and following up. One year later, she is totally independent and in good health, and her echocardiography showed normal ejection fraction.

### Risk factors of IAAD

IAAD after cardiac surgery is a rare and devastating complication. Here we present a case of successful management after very complicated clinical course. Cannulation of the ascending aorta for CPB was first announced in 1959 [2], and since then, IAAD continues to be a fearful fatal complication that turns routine open cardiac surgery into a major clinical challenge to the team who faces it.

Our patient had three risk factors: hypertension, aortic regurgitation and thinned dilated aorta, which we believe predisposed her to IAAD. In a recent editorial in which IAAD was described as a perfect storm, the author suggested to consider replacement of the ascending aorta in patients having these three risk factors [1].

Other risk factors for IAAD include old age, known atherosclerotic disease and previous CABG surgery [3]. In addition, high and low blood pressures during insertion of the aortic cannula play a role in the development of IAAD [4]. Very low blood pressure renders the aortic wall unsupported during cannulation with increased liability to dissection.

### Recent classification for IAAD in cardiac surgery

Recently, in a multicenter study by Stanger et al. [5], IAAD following cardiac surgery is classified into *intraoperative*, *early postoperative* (symptoms of aortic dissection within 30 days of surgery with high risk of aortic rupture and intraoperative mortality) and *late postoperative* (after 30 days from cardiac surgery). They further classified late postoperative IAAD into *acute* (patients required intervention within 2 weeks of symptoms) and *chronic* (late incidental finding of dissection with no acute symptoms, candidates for elective surgery) (Table 1).

**Table 1:** Trends in sites of iatrogenic aortic dissection after cardiac surgery in literature

	Gott et al. [10] (*N* = 27/11,145) 1982–1988	Still et al. [11] (*N* = 24/14,877) 1980–1990	Leontyev et al. [12] (*N* = 36/55,279) 1995–2010	Stanger et al. [5] (*N* = 103/68,249) 2006–2010
1- Intraoperative				
	*(N* = *27)*	*(N = 20)*	*(N = 31)*	*(N = 24)*
Aortic cannulation	10 (37 %)^a^	10 (50 %)^a^	12 (38 %)^a^	7 (29.1 %)^a^
Cardioplegia cannula	5 (18.5 %)	0 (0 %)	7 (22.6 %)	4 (16.7 %)
Aortic cross-clamp	4 (14.8 %)	8 (40 %)	4 (12.9 %)	7 (29.1 %)^a^
Proximal anastomosis	2 (7.4 %)	1 (5 %)	1 (3.2 %)	5 (20.8 %)
Direct aortic injury	0 (0 %)	1 (5 %)	0 (0 %)	0 (0 %)
Unknown	2 (7.4 %)	0 (0 %)	5 (16.1 %)	1 (4.2 %)
2- Early Postoperative:				
		*(N* = *4)*	*(N = 5)*	*(N = 12)*
Aortic cannulation		3 (75 %)^a^	2 (40 %)^a^	0 (0 %)
Cardioplegia cannula		0 (0 %)	0 (0 %)	1 (8.3 %)
Aortic cross-clamp		1 (25 %)	0 (0 %)	1 (8.3 %)
Proximal anastomosis		0 (0 %)	1 (20 %)	8 (66.7 %)^a^
Aortotomy		0 (0 %)	0 (0 %)	2 (16.7 %)
Unknown		0 (0 %)	2 (40 %)^a^	0 (0 %)
3- Late: Postoperative				
a) Acute				*(N = 44)*
Aortic cannulation				1 (2.2 %)
Cardioplegia cannula				0 (0 %)
Aortic cross-clamp				2 (4.5 %)
Proximal anastomosis				10 (22.7 %)
Aortotomy				24 (54.5 %)^a^
Unknown				7 (15.9 %)
b) Chronic				(*N* = 23)
Aortic cannulation				2 (8.7 %)
Cardioplegia cannula				0 (0 %)
Aortic cross-clamp				0 (0 %)
Proximal anastomosis				9 (39.1 %)^a^
Aortotomy				11 (47.8 %)
Unknown				1 (4.3 %)

^a^Represents highest incidence in each category within every study

### Incidence and common entry sites of IAAD

Literature shows the incidence of IAAD to be 0.12–0.23 %; mortality rates range from 20 to 43 % [6, 7], with decreased rates when IAAD is diagnosed intraoperatively, and its extension is limited to the aortic arch [8].

Most dissection sites mentioned in the literature after open cardiac surgery were related to aortic cannulation, aortic cross-clamping, aortotomy, vein anastomosis during CABG, the cardioplegia cannula, direct aortic injury and injury related to flow of the bypass to the aortic arch or descending thoracic aorta [5, 9–12]. Reviewing the literature in 3 single center and 1 multicenter studies showed nearly the same trends in sites of IAAD over that last 35 years (Table 1) [5, 10–12]. In the majority of cases, the site of dissection in intraoperative cases was the cannulation territory which is consistent with our case.

### Prevention and management of IAAD

#### Role of surgeons

Aortic cannulation is the single most important step in cardiac surgery and prevention of IAAD is the best treatment strategy. Surgeons should be extremely cautious during aortic cannulation, clamp application and removal especially in patients with risk factors for IAAD. Cannulation purse‑string sutures should be taken with deep partial or full thickness bites. Surgeons should also avoid torsion and proximity of the partial‑occlusion clamp to the aortic cannula [13].

IAAD diagnosis requires high index of suspicion and it depends primarily on visualization and imaging. Intraoperative diagnosis is achieved by visualization of a bluish discoloration and dilatation of the aorta [9]. Still et al. [11] differentiated a subadventitial hematoma from IAAD; the subadventitial hematoma is usually small, enlarges slowly, stops enlarging after initiation of bypass, soft to palpation and can be decompressed by incising the aortic surface. IAAD, on the other hand, may involve the entire aorta or part of it, giving it a tense, dilated bluish discoloration, and bleeds rapidly on incision of the adventitial surface. Once IAAD is confirmed, the surgeon has to secure an alternative peripheral site of cannulation and ensures that the blood flow is through the true aortic lumen.

#### Role of anesthesiologists

Anesthesiologists are encouraged to routinely examine the cannulation site after insertion and removal of the aortic cannula as well as the other previously reported possible entry sites of dissection whenever possible using TEE. Routine use of TEE aids in the rapid diagnosis and the prompt management of IAAD with a sensitivity of 98 % and specificity of 95 %. TEE also differentiates dissection from subadventitial hematoma. However, visualization of IAAD by TEE in the distal ascending aorta and proximal arch may be limited due to the interposition of tracheal air between the ultrasonic probe and the aorta, and may require an epiaortic echocardiographic probe [6, 14, 15]. Moreover, epiaortic scanning can be used when there is a contraindication for the use of TEE [16]. Once IAAD is confirmed and until an alternate cannulation site is secured, the anesthesiologists have an important role to support the circulation during the transition from CPB to spontaneous circulation in order to maintain critical organs perfusion.

#### Role of perfusionist

Communication between the perfusionist and the surgeon is mandatory during the test transfusion to ensure proper placement of the aortic cannula. The pump should be started slowly, while the arterial line pressure is being checked for any pressure rise denoting obstruction. Aortic dissection is among one of the causes of abrupt increase in the arterial pressure line and needs to be excluded. It is advisable for the perfusionist to reduce the pump flow (0.5–1 L/min) during insertion or removal of the aortic cannula [17].

After diagnosis of IAAD, the underlying cause should be corrected and the management plan should be changed aiming at repair of the dissection. In cases with small intimal tear, simple primary repair could be achieved [18]. In our case, we had to initiate CPB through peripheral cannulation and performed hypothermic circulatory arrest for repair of the dissection. The rate of institution of hypothermic circulatory arrest for repair of IAAD mentioned in the literature ranged 50–95 % [5, 10–12].

Ruchat et al. [9] mentioned that cerebral malperfusion is one of the unfavorable outcomes of IAAD that may be due to extension of the dissection beyond the aortic arch. In their study, two of seven patients who experienced IAAD died of hypoxic brain insult. Prompt intraoperative diagnosis and management of IAAD may prevent this sequel. In another study, stroke was reported in 27 of 103 patients with IAAD [5]. Our patient developed right middle cerebral artery infarction that was most probably due to cerebral hypoperfusion from severe hemodynamic instability or embolism, but was not explained to be due to vascular dissection neither by intraoperative evidence through direct visualization of the arch vessels or cerebral monitoring, nor by postoperative imaging using carotid Doppler and MRA.

Our patient had a complicated postoperative course in the ICU, including bleeding and recurrent cardiac tamponade. After one year follow up, our patient, is in good health owing to the team approach.

### ECMO support and coagulation management

The use of ECMO as a salvage modality for cardiogenic shock, specifically post-cardiotomy cardiogenic shock, may offer advantages over conventional medical therapies [19, 20]. The benefit of mechanical support providing improved cardiac output without the negative hypoperfusion consequences, led to the proactive initiation of VA ECMO support. Squiers et al. [21] concluded that VA ECMO offers certain advantage in rare instances of postcardiotomy shock, in addition to other studies that showed excellent long-term survival in patients surviving the acute perioperative period.

Our patient required transfusion of 140 units of blood and blood products in a very short period of time. Massively transfused patients have improved survival when receiving a high fresh frozen plasma:RBC and platelet:RBC ratio [22]. As such, we utilize point of care testing rotational thromboelastometry (ROTEM) perioperative in our patients, which leads to an increase in fresh frozen plasma and platelet to RBC transfusion ratio, that is consequently associated with improved survival [22].

Our center utilizes Extracorporeal Life Support Organization (ELSO) guidelines for anticoagulation and therapeutic management of unfractionated heparin (UNFH) during mechanical support. [20] We utilize an ECMO circuit with a biocompatible coated surface that provides a significant benefit in patients with massive coagulopathic bleeding, as it allows the delay of anticoagulation at the start of extracorporeal life support (ECLS).

The activated clotting time (ACT) is a universally accepted, and a readily available intraoperative point of care test to assess and dose UNFH. However, post-operative therapeutic anticoagulation, will be influenced by many factors including urine output, renal replacement therapy, platelet administration, and underlying coagulation changes. Additionally, the ACT test will be affected by postoperative factors such as thrombocytopenia, and smaller percent changes in individual clotting cascade factors than the activated partial thromboplastin time (APTT). A recent study in pediatric ECLS patients showed that the APTT (clinical laboratory and POC) correlated to UNFH dose better than ACT and, as expected the APTT and UNFH dose correlation improved with increasing patient age [23]. As recommended per ELSO guidelines, using one method of UNFH activity alone is considered insufficient today, and yet an array of too many tests for UNFH therapy, multiple times per day, could provide possible confusion to ECLS team members [20]. Our center therefore monitors UNFH activity during the postoperative course with the more sensitive APTT test, to maintain a therapeutic level, and supplementation of ROTEM tests as needed.

### IAAD in other minimally invasive cardiovascular procedures

IAAD can present with other cardiovascular procedures: during diagnostic and therapeutic coronary artery intervention, TEVAR and TAVR. The clinical presentation and the management vary widely.

#### IAAD during coronary artery intervention

In the Catheterization Lab, IAAD may occur during diagnostic and therapeutic PCI. The incidence of IAAD in cardiac catheterization is less than 0.1 %. It may involve the coronary arteries and/or the aorta. National, Heart, Lung and Blood Institute (NHLBI) classified coronary artery dissection to 6 types, none of which involved the aortic root [24]. A simplified classification for this type of dissection was mentioned by Eshtehardi et al. [25]: *type I* (localized dissection in the coronary arteries), *type II* (extension of dissection into major coronary branches), and type III (extension of the dissection into the aortic root). Cardiac surgeons will manage emergency cardiac surgeries in a small fraction of cases of cardiac catheterization complicated by IAAD. Current data on incidence and the clinical outcome of IAAD in cardiac catheterization is limited. In multicenter study the incidence of IAAD was 0.062 %, of those involving the coronary tree was 0.039 % with higher incidence in therapeutic than in diagnostic procedures. The operator expertise and the use of guiding wires in complex procedures are suggested risk factors leading to IAAD after PCI [26].

In a study by Leontyev et al. [12], 12 out of 135,262 patients developed IAAD after cardiac catheterization; all patients underwent surgical intervention with 30 day mortality of 50 % denoting high mortality in this group of patients. In another registry, only 3 patients out of 74 patients with IAAD after PCI were referred to surgery. The rest of the patients were managed either conservatively or by PCI [26]. This might be attributed to difference in local practice and/or the type and extent of dissection. In patients with retrograde dissection not involving the coronary arteries, conservative management may be attempted together with close patient monitoring for possible surgical intervention. On the other hand, antegrade dissections usually require endovascular or surgical management [26].

#### IAAD during TEVAR

Minimal invasive techniques have changed clinical practice for management of thoracic aortic diseases. TEVAR is the first choice therapy for most thoracic aortic disease. IAAD after TEVAR was also reported in the literature with incidence ranging 1–6.8 % [27, 28]. Several factors leading to this complication have been proposed: instrumentation and stent negotiation, underlying aortic pathology (e.g. aneurysm, ulcer) leading to fragile wall, oversized grafts, excessive aortic arch angulation and expanding intramural hematoma [29, 30]. Even with immediate open surgical management to prevent grave outcomes, mortality rate in the literature is 44–57 % [30].

#### IAAD during TAVR

With the rapid increase in the number of TAVR cases around the globe, the association between IAAD and TAVR is also emerging in the literature. IAAD after TAVR procedure is mentioned as part of the major vascular complications. The incidence ranges from 0.2 % in the GARY registry (33 IAAD of 15,964 patients) to 2 % in transapical TAVR cases in another review [31, 32]. In the PARTNER trial, there were 3 patients with IAAD out of 419 included in the trial; however, in the three studies, specific clinical management of IAAD was not mentioned [33].

## Conclusion

IAAD is a dreadful complication that carries high morbidity and mortality. All cardiovascular professionals should be ready to diagnose and quickly intervene specially with the increase in numbers and complexity of minimally invasive procedures.

## Abbreviations

ACT: Activated clotting time
APTT: Activated partial thromboplastin time
AVR: Aortic valve replacement
CPB: Cardiopulmonary bypass
ECLS: Extracorporeal life support
ECMO: Extracorporeal membrane oxygenation
ELSO: Extracorporeal life support organization
IAAD: Iatrogenic acute aortic dissection
NHLBI: National, heart, lung and blood institute
PCI: Percutaneous coronary intervention
ROTEM: Rotational thromboelastometry
TAVR: Transaortic valve replacement
TEE: Transesophageal echocardiography
TEVAR: Thoracic endovascular aortic repair
UNFH: Unfractionated heparin
VA ECMO: Venoarterial Extracorporeal membrane oxygenation
